# Application research of “Script Killing” immersive teaching method based on cross-experimental design during emergency department residency training rotations

**DOI:** 10.1371/journal.pone.0355170

**Published:** 2026-08-03

**Authors:** Dalong Zhang, Xingguo Niu, Zhenguan Lei, Can Lyu, Jingyang Lyu, Shengjie Huo, Yanan Liu, Juhua Tang, Jiankai Gao

**Affiliations:** 1 The Fifth Clinical Medical College of Henan University of Chinese Medicine (Zhengzhou People’s Hospital), Zhengzhou, China; 2 Henan University of Chinese Medicine, Zhengzhou, China; 3 Emergency Department of Zhengzhou People’s Hospital, Zhengzhou, China; University of Marburg: Philipps-Universitat Marburg, GERMANY

## Abstract

In order to evaluate the effectiveness of the “Script Killing” teaching method and the traditional teaching method in enhancing the learning outcomes of emergency medicine resident trainees during the standardized training period, from May to October 2024, we recruited 30 resident physicians from the emergency department of Zhengzhou People’s Hospital as the research subjects and randomly divided them into Group A and Group B. The experiment adopted a crossover design: the first stage (6 weeks) – Group A received traditional lecture-based teaching, while Group B received immersive Script Killing” teaching; the second stage (6 weeks) – the two groups exchanged teaching intervention methods. The assessment contents included satisfaction surveys, theoretical knowledge tests, practical skill evaluations, and patient-doctor evaluations. The experimental results were analyzed using SPSS 22.0 to adjust for confounding factors. The results showed that the satisfaction of the “Script Killing” group was significantly higher (P < 0.05), the theoretical scores were better (P < 0.05), and the practical performance was also better (P < 0.05), while there was no significant difference in the traditional teaching group. No statistical differences were observed in patient-doctor feedback. These preliminary findings suggest that the “Script Killing” teaching method may effectively enhance the clinical operational abilities and learning satisfaction of emergency medicine trainees. Although limited by a small sample size and single-center design, this approach shows promise as a beneficial supplement to standardized residency training, warranting further validation in larger cohorts.

## Introduction

Standardized Residency Training (SRT) serves as a critical pathway for transforming clinical medicine graduates into competent physicians. In an effort to enhance the quality of SRT, various innovative pedagogical models—such as Problem-Based Learning [1], case-based learning [2], and scenario simulation [3].—have been introduced and implemented.

The unique characteristics of the emergency department—including its inherent unpredictability, clinical complexity, and time-critical nature—often make it challenging for instructors to provide real-time, bedside teaching for critical cases. Traditional didactic methods, which primarily rely on PPT-based case lectures, offer limited avenues for knowledge acquisition. Consequently, trainees often struggle to develop the necessary clinical responsiveness for emergency settings, a competency that SRT is designed to foster. Therefore, identifying a teaching method that immerses trainees in simulated clinical emergencies is imperative.

Traditional situational simulation teaching, as described in the context of resident training, typically relies on standardized scenarios, simulated patients, or pre-set procedural protocols. While this approach has been shown to effectively train trainees in operational skills and stepwise clinical responses, it is often limited by relatively static scenarios, pre-scripted interactions, and a focus on individual or single-task performance [3]. These limitations mean that such simulations may not fully replicate the dynamic, unpredictable nature of real-world clinical encounters.

In contrast, immersive script-based teaching centers on narrative-driven, dynamic role-playing. Participants must navigate unpredictable variables introduced by non-player characters (NPCs)—such as family members refusing to provide consent, or sudden changes in the patient’s condition—rather than following fixed, linear procedures. Unlike traditional simulation, which often treats team roles as secondary, script-based teaching explicitly defines hierarchical team roles and responsibilities. This design directly fosters collaborative dynamics and reduces the “bystander effect” common in passive, single-task simulations.

Kavanaugh et al. first explored this type of narrative-driven, role-playing teaching in a pharmacy skills course, designing a “hospital murder mystery” activity in which students worked in teams to analyze clinical clues, identify potential causes of a simulated patient’s death, and propose improvements to clinical workflows [4]. The study demonstrated that this format effectively enhanced students’ critical thinking and team collaboration. The strong interactivity and exploratory nature of “Script Killing” align perfectly with the requirements of the emergency department, which constantly needs to search for clues to quickly diagnose complex conditions and promptly carry out rescue measures [5]. At present, there is a lack of systematic research on the application of this method in the emergency department residency training. The teaching effect of this method lacks empirical data support. Whether it can effectively enhance the theoretical level, practical skills and humanistic quality of the trainees still needs to be verified. To establish a rigorous pedagogical foundation, the “Script Killing” intervention was systematically conceptualized and developed based on a dual theoretical framework comprising Kolb’s Experiential Learning Theory [6] and Self-Determination Theory [7], embedded within a social constructivist learning environment [8]. Within this study, the narrative-driven mechanics of the scripts were designed to guide emergency medicine trainees through a structured cognitive cycle of concrete experience and active experimentation [6], while the inherent gamified elements [9] targeted the cultivation of their intrinsic educational motivation [7].The overall goal of this study is to conduct a cross-experimental design to compare the application effects of the “Script Killing” immersive teaching method and the traditional teaching method in the training of emergency department residents. To clarify the impact of this innovative teaching method on the satisfaction of trainees, the mastery of theoretical knowledge, the improvement of practical skills and the communication ability with patients.

## Materials and methods

Due to the small number of research subjects, in order to ensure the test efficacy, a randomized crossover design was adopted. Considering the educational differences caused by the allocation, which led to different educational experiences among different students and resulted in unfair educational distribution, the cross-experimental design commonly used to explore the efficacy of drugs was selected.

### Subjects

Using a convenience sampling technique, thirty resident trainees in the Emergency Department of Zhengzhou People’s Hospital were recruited between May and October 2024, including 14 males and 16 females. Inclusion criteria: (1) Voluntary participation in this study; (2) Participants agreed to the teaching methods used; and (3) Detailed records of emergency medicine teaching and evaluation data were available. Exclusion criteria: (1) Failure to complete the entire emergency department rotation; (2) Personnel transfers preventing completion of follow-up research. All participants were stratified by gender and randomly divided into Group A and Group B, with 7 males and 8 females in each group. Their detailed demographic profiles, including age, are presented in Table 1.

**Table 1:** General information of the two groups.

	Group A	Group B	statistic	P-value
AGE	25.17 ± 0.53	25.35 ± 0.49	t = 0.97	0.34
Gender (M/F)[n(%)]	7(46.7)/8(53.3)	7(46.7)/8(53.3)	χ²=0.00	1.00
Ethnicity (Han)[n(%)]	15(100)	14(93.3)	χ²=1.03	0.309
The only child (is)[n(%)]	6(40.0)	5(33.3)	χ²=0.14	0.69
Place of residence (rural/urban)[n(%)]	5(33.3)/10(66.7)	6(40.0)/9(60.0)	χ²=0.14	0.69

### Research methods

Following grouping, the subjects underwent two distinct six-week teaching interventions: traditional teaching and the “Script Killing” teaching method. After the first six weeks, the two teaching methods were swapped between groups for a subsequent six-week period [10,11]. Specifically:

Phase I: Group A received the traditional teaching method, while Group B underwent the “Script Killing” teaching method.

Phase II: Group A switched to the “Script Killing” teaching method, and Group B switched to the traditional teaching method.

Based on the teaching syllabus and the actual work situation, selected common and error-prone diseases such as male respiratory and digestive system diseases as teaching examples.

**Fig 1:**
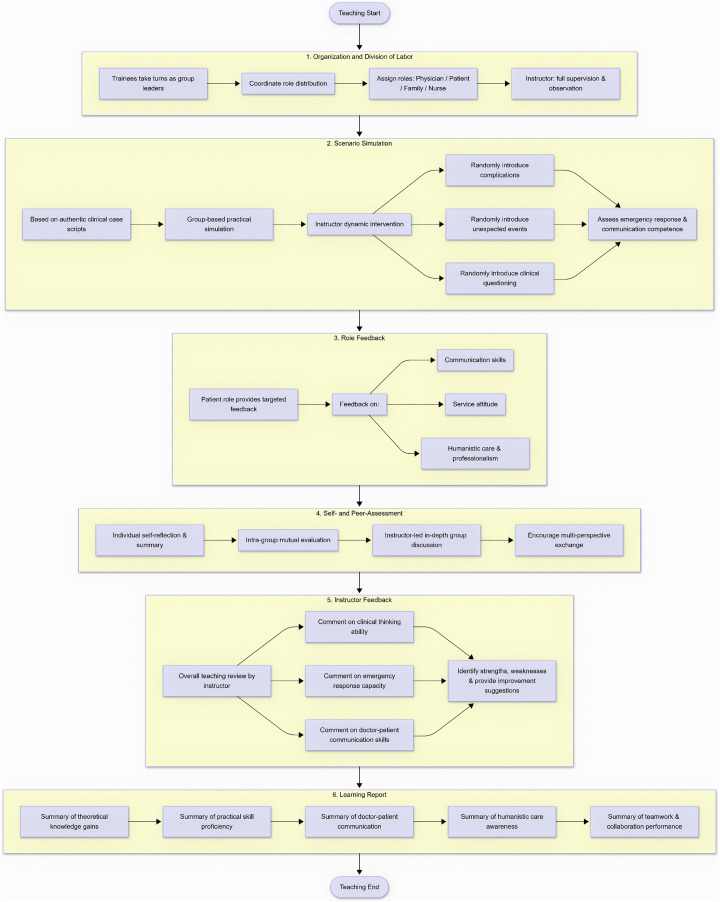
“Script Killing” teaching protocol.

“Script Killing” Teaching Protocol (Fig 1):

1. Organization and Division of Labor: For each session, group members took turns serving as the team leader, responsible for coordinating the role-play. Members assumed various roles as required by the scenario—such as physician, patient, family member, nurse, or other departmental staff—to realistically replicate clinical settings. Instructors observed and provided guidance when necessary. Trainees were permitted to make minor adjustments to the scenario to enhance authenticity and engagement, provided they adhered to clinical realism.
2. Scenario Simulation: Groups conducted practical drills according to the prepared cases. Instructors could introduce unexpected complications or pose clinical questions during the simulation to assess trainees’ on-the-spot response and communication skills.
3. Role Feedback: Trainees playing the patient role provided feedback to those playing the physician role regarding their communication, attitude, and humanistic care.
4. Self- and Peer-Assessment: Following the simulation, group members conducted self- and peer-evaluations. Instructors facilitated in-depth discussions, encouraging the exchange of diverse perspectives.
5. Instructor Feedback: Instructors summarized the entire simulation, focusing on analyzing trainees’ clinical reasoning, emergency response capabilities, and communication skills. Strengths and weaknesses were highlighted, along with suggestions for improvement.
6. Learning Report: Trainees summarized their gains in theoretical knowledge, procedural skills, doctor-patient communication, humanistic care, and teamwork abilities.

**Fig 2:**
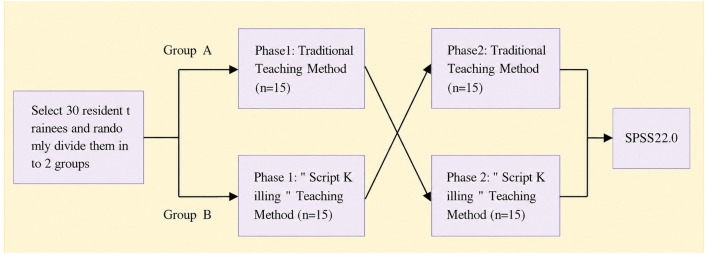
Randomized crossover experiment procedure.

Traditional Teaching Protocol: The same cohort of instructors conducted sessions following the conventional lecture-based teaching model. The curricular content was identical between the two methods and across both phases for each group. Assessments were conducted after each teaching session to evaluate and compare the effectiveness of the “Script Killing” and traditional teaching methods (Fig 2).

The study was approved by the Medical Ethics Committee of Zhengzhou People’s Hospital (Ethics Review No. 2005-ZYLW-007). All participants provided written informed consent prior to enrollment. The study used only de-identified data collected during routine training activities, with no additional identifiable information recorded or analyzed.

### Evaluation metrics

A self-developed questionnaire was utilized to investigate the effectiveness of the immersive “Script Killing” teaching model. Specific evaluation items included trainee satisfaction with the instruction, theoretical examination scores, practical skills examination scores, and doctor-patient relationship evaluations.

1. Trainee Satisfaction Evaluation: For this study, a self-designed satisfaction questionnaire was developed, covering four dimensions: teaching attitude, teaching content, teaching methods, and teaching effect. Each dimension had 5 items, making a total of 20 items. A 5-point Likert scale was used, with a maximum score of 25 for each dimension and a total score of 100. The survey data were entered and analyzed using the “Education and Science Assistant” evaluation software (The hospital’s proprietary internal assessment system for regular resident physicians). The content validity of this self-designed questionnaire was rigorously evaluated and confirmed by an expert panel comprising three senior emergency medicine educators prior to administration. Additionally, reliability analysis demonstrated good internal consistency, yielding a Cronbach’s α coefficient of 0.742.
2. Theoretical Examination Scores: The theoretical examination was closely aligned with the Standardized Residency Training syllabus. Instructors developed the questions based on key knowledge points related to the taught diseases. The exam consisted of 50 multiple-choice questions (2 points each), totaling 100 points. It was administered as a computer-based online test in the dedicated computer room of the hospital’s Education Office, with a time limit of 1 hour. The process was proctored throughout by Education Office staff to ensure strict adherence to examination protocols and fairness. Any identified cases of cheating led to the exclusion of the involved trainee’s data. Examination scores were anonymized using coded identifiers, collected, and entered into the database by research team members.
3. Practical Skills Examination Scores: The skills examination was conducted following the theoretical test. It simulated the national standardized residency completion skills examination process, adopting a multi-station assessment model to comprehensively evaluate trainees’ practical operational abilities. All examiners were independent certified provincial skill examiners who had not been involved in the teaching sessions, ensuring independent and professional scoring. The assessment took place in the hospital’s Clinical Skills Training Center, with a total score of 100 points based strictly on operational scoring criteria, preventing any manual intervention or cheating. Any identified cheating resulted in data exclusion. Scores were anonymized via coded identifiers, collected, and entered into the database by the research team.
4. Doctor-Patient Relationship Evaluation: To assess trainee performance in realistic clinical interactions, a two-way satisfaction evaluation mechanism was introduced. This included feedback from the physician on the patient’s attitude and cooperation, and from the patient on the physician’s service attitude and consultation experience. The total evaluation score was set at 10 points, reflecting the subjective experience and trust established during the interaction and serving as an indicator of humanistic communication and empathetic skills.

### Statistical methods

Statistical analysis was performed using SPSS 22.0. Quantitative data were described using the mean ± standard deviation (x̄ ± s), and group comparisons were conducted using t-tests. Qualitative data were described using frequencies and percentages, with group comparisons made via chi-square (χ²) tests. Data from the crossover trial design were analyzed using analysis of variance (ANOVA) to compare effects across different sequences, interventions, and periods. A P-value < 0.05 was defined as statistically significant. To quantify the practical significance of the observed differences beyond statistical significance, Cohen’s d was calculated as the standardized effect size for all t-test comparisons. According to Cohen’s conventions, d values of 0.2, 0.5, and 0.8 were interpreted as small, medium, and large effects, respectively.

## Results

### Comparison of baseline characteristics between groups

Demographic characteristics, including age, gender distribution, ethnicity, single-child status, and place of residence, were collected for both Group A and Group B. The mean age of Group A was 25.17 ± 0.53 years, and that of Group B was 25.35 ± 0.49 years. Statistical results indicated no significant differences in any of the demographic indicators between the two groups (P > 0.05), as shown in Table 1. This demonstrates good inter-group balance in the sample, effectively reducing the impact of potential confounding factors.

### Comparison of satisfaction scores between groups

Following the validation of the self-developed satisfaction questionnaire described in the methods section, the post-instruction satisfaction survey revealed that the phases employing the “Script Killing” teaching method received significantly higher scores across all four dimensions—instructor attitude, content design, teaching method, and instructional effectiveness—compared to the phases employing the traditional teaching method(all P < 0.001) (Table 2 and Table 3).

**Table 2:** Intra-group comparison of satisfaction scores before and after intervention.

	Group A (Phase I, n = 15)	Group A (Phase II, n = 15)	Comparison (t/P), Cohen’s d	Group B (Phase I, n = 15)	Group B (Phase II, n = 15)	Comparison (t/P), Cohen’s d
Teaching Attitude	18.8 ± 1.2	20.7 ± 1.1	t=4.02, P=0.001 d = 1.65	20.8 ± 1.1	18.7 ± 1.3	t = 4.15, P < 0.001 d = 1.74
Teaching Content	18.7 ± 1.3	20.5 ± 1.2	t=3.96, P=0.001 d = 1.44	20.6 ± 1.2	18.5 ± 1.3	t = 4.08, P < 0.001 d = 1.68
Teaching Methods	17.7 ± 1.5	21.6 ± 4.0	t = 6.85, P < 0.001 d = 1.29	21.5 ± 1.1	17.5 ± 1.5	t = 7.02, P < 0.001 d = 3.04
Teaching Effectiveness	18.5 ± 1.4	20.6 ± 1.1	t = 4.18, P < 0.001 d = 1.67	20.7 ± 1.2	18.3 ± 1.4	t = 4.25, P < 0.001 d = 1.84
Total Score	73.7 ± 4.8	83.4 ± 3.8	t = 5.62, P < 0.001 d = 2.24	83.6 ± 4.2	73.0 ± 5.0	t = 6.18, P < 0.001 d = 2.30

**Table 3:** Inter-group comparison of satisfaction scores in the same phase.

	Group A (Phase I, n = 15)	Group B (Phase I, n = 15)	Comparison (t/P), Cohen’s d	Group A (Phase II, n = 15)	Group B (Phase II, n = 15)	Comparison (t/P), Cohen’s d
Teaching Attitude	18.8 ± 1.2	20.8 ± 1.1	t = 4.60, P < 0.001 d = 1.74	20.7 ± 1.1	18.7 ± 1.3	t = 4.60, P < 0.001 d = 1.67
Teaching Content	18.7 ± 1.3	20.6 ± 1.2	t = 4.28, P < 0.001 d = 1.52	20.5 ± 1.2	18.5 ± 1.3	t = 4.37, P < 0.001 d = 1.48
Teaching Methods	17.7 ± 1.5	21.5 ± 1.1	t = 7.12, P < 0.001 d = 2.89	21.6 ± 4.0	17.5 ± 1.5	t = 3.84, P < 0.001 d = 2.81
Teaching Effectiveness	18.5 ± 1.4	20.7 ± 1.2	t = 4.64, P < 0.001 d = 1.69	20.6 ± 1.1	18.3 ± 1.4	t = 4.76, P < 0.001 d = 1.64
Total Score	73.7 ± 4.8	83.6 ± 4.2	t = 6.05, P < 0.001 d = 2.20	83.4 ± 3.8	73.0 ± 5.0	t = 6.52, P < 0.001 d = 2.14

**Fig 3:**
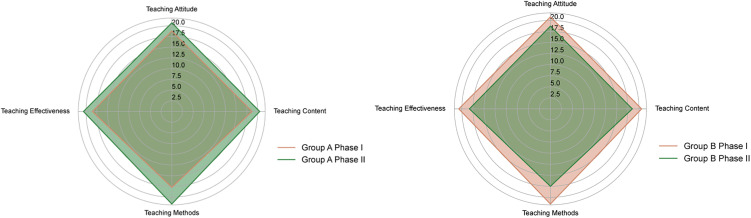
Intra-group comparison of satisfaction scores before and after intervention.

**Fig 4:**
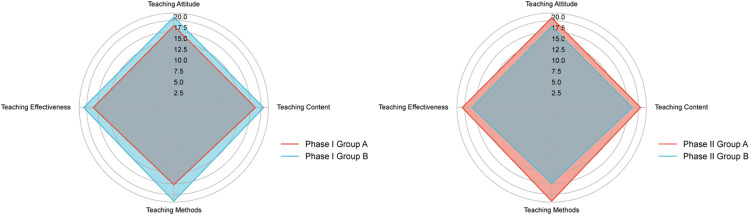
Inter-group comparison of satisfaction scores in the same phase.

In within-group comparisons (Table 2), Group A showed significant improvements in satisfaction across all dimensions after receiving “Script Killing” teaching, with the most pronounced increase observed in the teaching method dimension. Conversely, Group B (which received “Script Killing” first followed by traditional teaching) exhibited significant decreases in all satisfaction scores after switching to the traditional method. In between-group comparisons during the same period (Table 3), the group using the “Script Killing” method consistently achieved significantly higher satisfaction scores than the group using the traditional method, in both Phase I and Phase II(all P < 0.001) (Figs 3 and 4).

### Theoretical and practical skills examination scores

Trainees’ performance scores further validated the advantage of the “Script Killing” teaching method.

**Fig 5:**
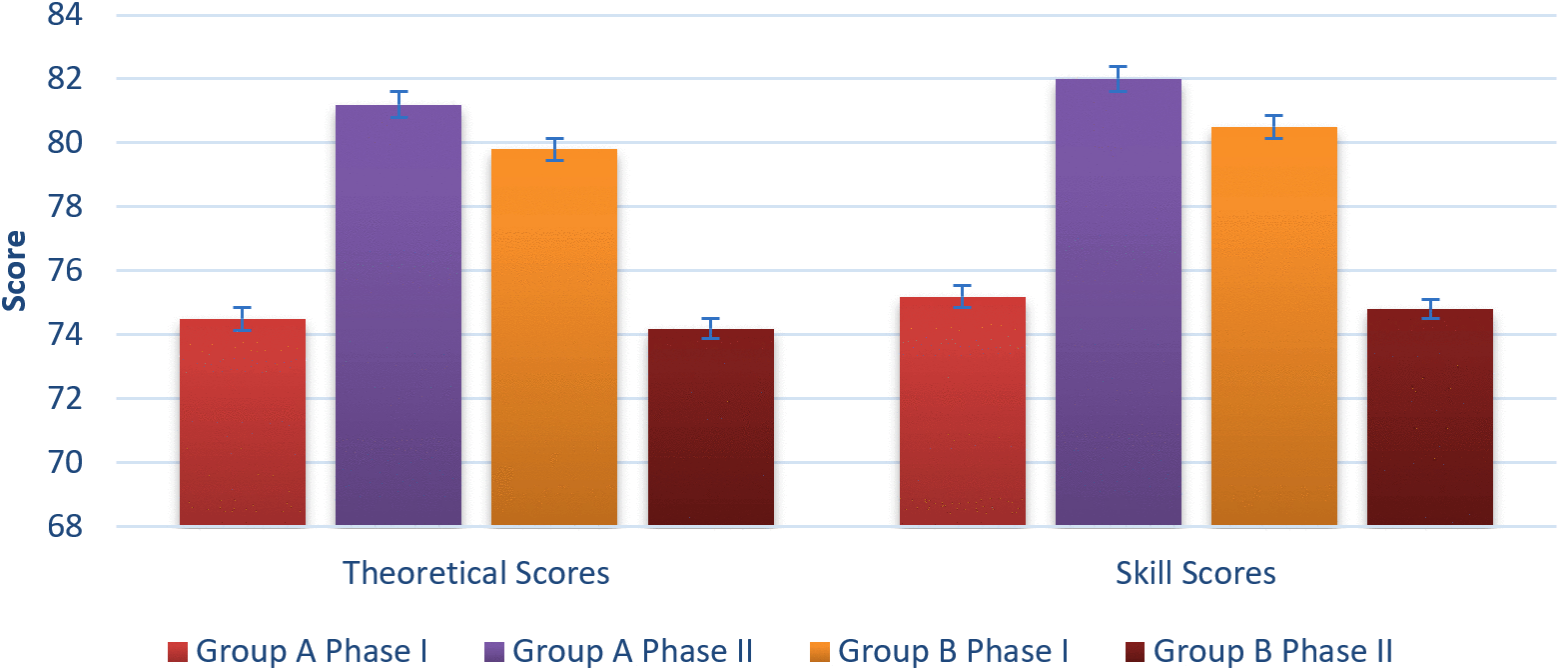
Theoretical and skill scores of both groups across two phases.

In within-phase comparisons (Table 4), Group A demonstrated significant improvements in both theoretical and practical skills scores after receiving “Script Killing” teaching. In contrast, Group B showed significant declines in both theoretical and practical scores after switching to the traditional method. In between-group comparisons during the same period (Table 5), during Phase I, Group B (using “Script Killing”) achieved significantly higher theoretical and practical scores than Group A (using traditional teaching)(both P < 0.05). During Phase II, Group A (using “Script Killing”) also achieved significantly higher theoretical and practical scores than Group B (using traditional teaching)(both P < 0.01) (Fig 5).

**Table 4:** Intra-group comparison of theoretical and skill exam scores.

Group	Score Type	Phase I	Phase II	t-value	P-value	Cohen’s d
Group A	Theory	74.5 ± 5.8	81.2 ± 5.5	3.82	0.002	1.19
Group A	Skill	75.2 ± 6.3	82.0 ± 6.0	3.65	0.002	1.11
Group B	Theory	79.8 ± 5.6	74.2 ± 5.7	3.78	0.002	0.99
Group B	Skill	80.5 ± 6.1	74.8 ± 6.2	3.59	0.003	0.93

**Table 5:** Inter-group comparison of theoretical and skill exam scores in the same phase.

	Score Type	Group A	Group B	t-value	P-value	Cohen’s d
Phase I	Theory	74.5 ± 5.8	79.8 ± 5.6	2.55	0.017	0.93
Phase I	Skill	75.2 ± 6.3	80.5 ± 6.1	2.34	0.027	0.86
Phase II	Theory	81.2 ± 5.5	74.2 ± 5.7	3.42	0.002	1.25
Phase II	Skill	82.0 ± 6.0	74.8 ± 6.2	3.23	0.003	1.18

To more rigorously analyze the crossover design data, analysis of variance (ANOVA) was conducted (Tables 6 and 7). The results showed a statistically significant effect of the teaching method (treatment effect) on performance scores (Theoretical score: F = 9.82, P < 0.05; Practical score: F = 8.84, P < 0.05), confirming that the “Script Killing” method was significantly superior to the traditional method in enhancing trainee performance. The effects of different trial sequences and different periods were not statistically significant, ruling out interference from learning order or time period on the main conclusions.

**Table 6:** ANOVA results for theoretical scores in the randomized crossover trial.

	Partial SS	df	Mean Square	F-value	P-value
Sequence Effect	9.8	1	9.8	0.52	0.47
Treatment Effect	186.5	1	186.5	9.82	0.004
Period Effect	15.2	1	15.2	0.81	0.37
Individual Effect	532.6	28	19.0	1.00	0.49

**Table 7:** ANOVA results for skill scores in the randomized crossover trial.

	Partial SS	df	Mean Square	F-value	P-value
Sequence Effect	11.5	1	11.5	0.59	0.45
Treatment Effect	172.3	1	172.3	8.84	0.006
Period Effect	13.8	1	13.8	0.71	0.40
Individual Effect	546.2	28	19.5	1.00	0.48

### Doctor-patient relationship evaluation

**Fig 6:**
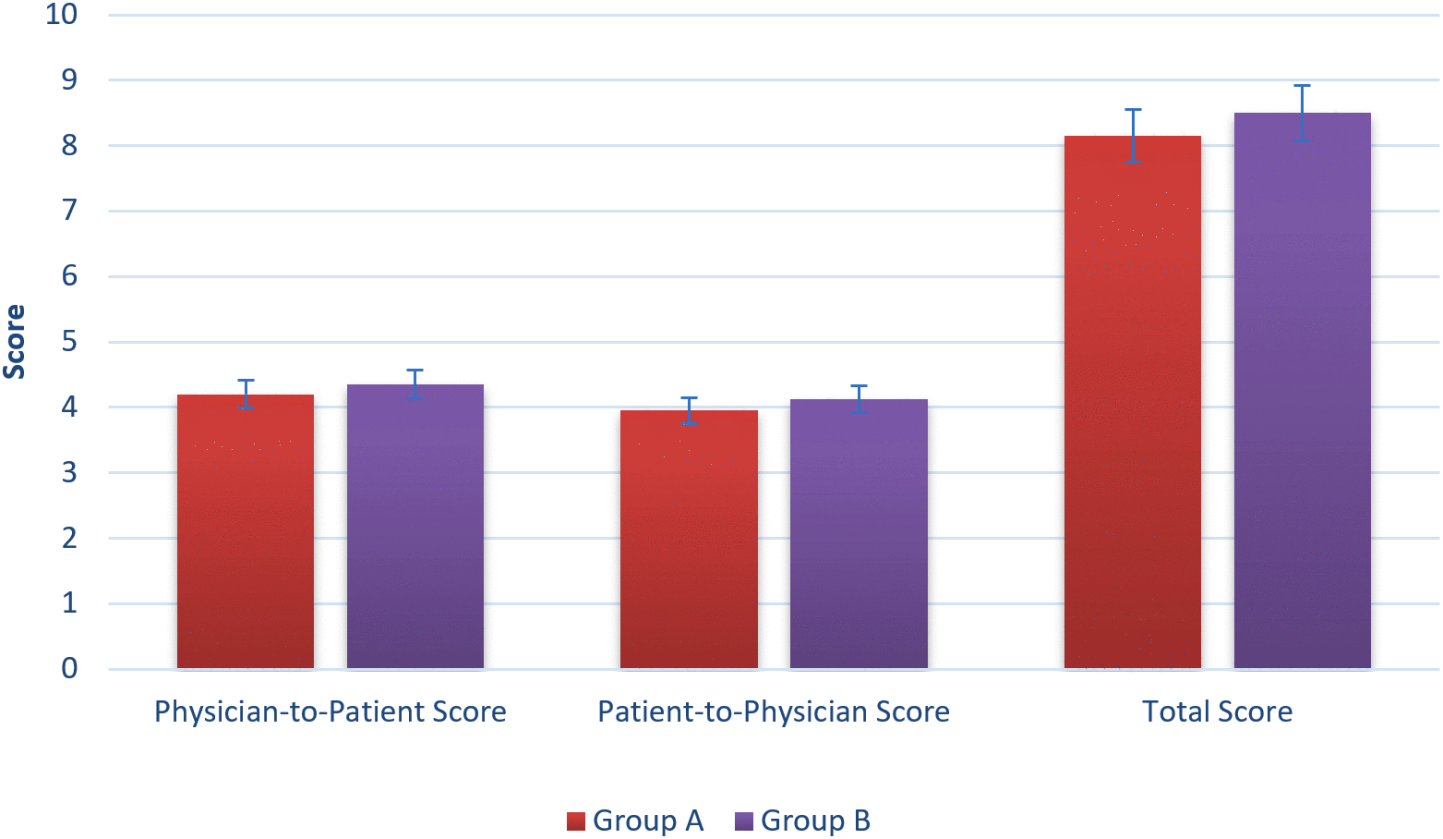
Physician-patient mutual evaluation scores during the “Script Killing” phase in both groups.

During the “Script Killing” teaching sessions, the mutual evaluation results between trainees playing the roles of doctors and patients showed high total scores for the doctor-patient relationship evaluation in both Group A and Group B (8.15 ± 1.60 and 8.50 ± 1.42, respectively), with no significant difference between the two groups (P = 0.416) (Table 8). These findings suggest that trainees achieved generally favorable doctor-patient interaction scores during the “Script Killing” sessions; however, no statistically significant differences were observed between groups (Fig 6).

**Table 8:** Mutual evaluation results of physician-patient relationship.

	Group A(Score)	Group B(Score)	Statistic t	P-value
Physician-to-Patient Score	4.20 ± 0.95	4.35 ± 0.82	0.51	0.612
Patient-to-Physician Score	3.95 ± 0.88	4.12 ± 0.75	0.78	0.439
Total Score	8.15 ± 1.60	8.50 ± 1.42	0.82	0.416

## Discussion

This study compared a theory informed “Script Killing” immersive teaching method with traditional lecture-based instruction in emergency medicine residency training. The results showed that the “Script Killing” method was significantly better at improving trainee satisfaction, theoretical knowledge, and practical skills. There was no significant difference between the two groups in doctor patient relationship scores. This immersive training has narrowed the gap between theoretical knowledge and the stressful real clinical environment. The effect sizes (Cohen’s d) for the “Script Killing” intervention ranged from 0.86 to 2.30 across all comparisons, indicating moderate to large practical effects that support the educational relevance of these findings. These findings suggest that the intervention may better prepare trainees for managing complex emergency situations and may contribute to improved clinical performance in future practice.

Beyond statistical significance, the observed improvements also appear to be educationally meaningful. Emergency medicine residency training emphasizes competency-based education, in which trainees are expected not only to acquire theoretical knowledge but also to integrate clinical reasoning, rapid decision-making, teamwork, and communication skills in complex clinical situations. The higher theoretical and practical examination scores observed in the “Script Killing” group suggest that participants may have developed a stronger ability to apply medical knowledge to authentic clinical scenarios. Furthermore, the immersive and collaborative nature of the intervention may better prepare residents for high-pressure emergency settings, thereby supporting the development of professional competencies that are essential for independent clinical practice.

The statistically significant advancements in both theoretical grasp and acute-care execution observed in the “Script Killing” group can be profoundly elucidated through these underlying theoretical constructs. Rather than undergoing passive instruction, trainees within the “Script Killing” cohort actively constructed clinical knowledge [8]. The high-fidelity, time-sensitive emergency scenarios provided the ‘concrete experience’ and ‘active experimentation’ stages of Kolb’s learning cycle, forcing immediate clinical reasoning under pressure. Concurrently, the structured post-game debriefing catalyzed ‘reflective observation’ [6], crystallizing localized simulation experiences into generalizable clinical pathways.

Furthermore, the superior satisfaction evaluations are tightly coupled with the motivational mechanics of the “Script Killing” format. According to Self-Determination Theory, the assignment of distinct professional roles and the requirement for collaborative diagnostic consensus directly satisfied the trainees’ core psychological needs for autonomy and relatedness [7]. This alignment successfully fostered high cognitive engagement and psychological authenticity [7,9], which are critical for clinical skill acquisition but frequently absent in traditional, lecture-based simulation modalities. Notably, the ANOVA results confirmed that these treatment effects were independent of sequence or period effects, reinforcing the robust reliability of these underlying pedagogical mechanisms within a rigorous crossover design [10,11].

These findings build on earlier work on game-based learning in medical education. Kavanaugh et al. showed that a murder mystery format was feasible in a pharmacy skills course [4], but their study used a single group design and did not compare it with traditional methods. The present study strengthens the evidence by using a controlled crossover comparison in a setting that has received little attention, namely emergency medicine residency training.

This study has certain limitations: Firstly, the small sample size (n = 30) and the single-center research design to some extent limit the external generalizability of the results. Secondly, due to the characteristic that the acquired knowledge in educational crossover trials cannot be “eliminated,” this study did not set up a formal washout period; although we implemented a strict “isolation strategy” by using two-stage completely independent and non-overlapping clinical cases and assessment question banks to prevent the transfer of specific knowledge, we still could not completely rule out the generalized transfer effect (although the statistical results did not show a significant sequential effect) caused by trainees’ familiarity with the simulation environment or improvement in overall clinical reasoning ability. Finally, the satisfaction questionnaire used in this study was developed independently, although it has been verified by an expert group for content validity and shows good internal consistency (Cronbach’s α = 0.742), it is still recommended to use more authoritative standardized assessment tools in future research. In conclusion, it is necessary to conduct large-scale, multi-center parallel group randomized controlled trials in the future to further verify and expand the findings of this study.

## Conclusion

This single-center crossover study provides preliminary evidence suggesting that the “Script Killing” immersive teaching method may enhance theoretical knowledge, practical skills, and learning satisfaction among emergency medicine residents when compared to traditional instruction. However, given the limitations of a small sample size and the single-center design, these findings should be interpreted with caution. Nevertheless, this gamified approach shows potential as a beneficial supplement to existing residency training models. Future large-scale, multi-center trials utilizing standardized assessment tools are necessary to confirm these preliminary results and establish their broader applicability.

## Supporting information

S1 File Clinical dataset of crossover experiment. (XLSX)

S2 File Teaching satisfaction questionnaire. (DOCX)

## References

[1] Ardoin TW, Hamer D, Stumpf M, Miles L. Integrating problem-based learning into an internal medicine residency curriculum. Ochsner J. 2022;22(4):324–43. doi: 10.31486/toj.22.0078 36561109 PMC9753947

[2] Zeng N, Lu H, Li S, Yang Q, Liu F, Pan H, et al. Application of the combination of CBL teaching method and SEGUE framework to improve the doctor-patient communication skills of resident physicians in otolaryngology department. BMC Med Educ. 2024;24(1):201. doi: 10.1186/s12909-024-05185-9 38413978 PMC10900716

[3] Shan T, Kejun W, Ying F, Jia H, Hongyan J. Efficacy and influencing factors of the four-step approach combining the situational simulation teaching method in the clinical practice of standardized training for residents. Health Sci Rep. 2022;5(5):e757. doi: 10.1002/hsr2.757 36101718 PMC9455944

[4] Kavanaugh R, Pape Z, LaTourette B, Lehmier S. Who killed Mr. Brown? A hospital murder mystery in a pharmacy skills course. Med Teach. 2022;44(10):1151–7. doi: 10.1080/0142159X.2022.2071690 35531595

[5] Lowe DJ, Ireland AJ, Ross A, Ker J. Exploring situational awareness in emergency medicine: developing a shared mental model to enhance training and assessment. Postgrad Med J. 2016;92(1093):653–8. doi: 10.1136/postgradmedj-2015-133772 27129912

[6] Kolb DA. Experiential learning: experience as the source of learning and development. 2nd ed. Upper Saddle River, NJ: Pearson Education; 2015.

[7] Ryan RM, Deci EL. Self-determination theory: basic psychological needs in motivation, development, and wellness. New York, NY: Guilford Press; 2017.

[8] Vygotsky LS. Mind in society: the development of higher psychological processes. Cambridge, MA: Harvard University Press; 1978.

[9] Deterding S, Dixon D, Khaled R, Nacke L. From game design elements to gamefulness: defining “gamification.” In: Proceedings of the 15th International Academic MindTrek Conference: Envisioning Future Media Environments. New York, NY: ACM; 2011. pp. 9–15. doi: 10.1145/2181037.2181040

[10] Murray E, Jolly B, Modell M. Can students learn clinical method in general practice? A randomised crossover trial based on objective structured clinical examinations. BMJ. 1997;315(7113):920–3. doi: 10.1136/bmj.315.7113.920 9361543 PMC2127606

[11] Offenbacher J, Petti A, Xu H, Levine M, Manyapu M, Guha D, et al. Learning outcomes of high-fidelity versus table-top simulation in undergraduate emergency medicine education: prospective, randomized, crossover-controlled study. West J Emerg Med. 2022;23(1):20–5. doi: 10.5811/westjem.2021.12.53926 35060855 PMC8782127

