# Supplementary material for: Application research of “Script Killing” immersive teaching method based on cross-experimental design during emergency department residency training rotations
Source: PLoS One. 2026 Aug 3;21(8):e0355170. doi: 10.1371/journal.pone.0355170 (PMC13432151; doi:10.1371/journal.pone.0355170)
Supplement: S2 File — (DOCX) [file pone.0355170.s002.docx]

**Questionnaire: Satisfaction Evaluation of the “Script Killing” Immersive Teaching Method**

**Instructions**: Please rate the following items based on your experience during the teaching sessions (5: Strongly Agree/Very Satisfied; 4: Agree/Satisfied; 3: Neutral; 2: Disagree/Dissatisfied; 1: Strongly Disagree/Very Dissatisfied).

**1. Teaching Attitude**

1. The instructor maintained a professional and enthusiastic teaching attitude throughout the session.

2. The instructor demonstrated a supportive approach toward trainee role participation.

3. The instructor provided timely and patient guidance when addressing clinical questions.

4. The instructor offered constructive and targeted feedback following the simulation.

5. The instructor effectively facilitated team communication and coordinated the teaching flow.

**2. Teaching Content**

6. The clinical scenarios were designed to realistically replicate complex emergency department settings.

7. The content selection was closely aligned with the standardized residency training syllabus.

8. The scenario design effectively facilitated the development of clinical logical thinking.

9. The inclusion of unexpected complications accurately tested my on-the-spot emergency response capabilities.

10. The teaching content successfully bridged the gap between theoretical knowledge and clinical practice.

**3. Teaching Methods**

11. The “Script Killing” immersive method is more interactive and engaging than traditional lecture-based teaching.

12. The division of roles (physician, patient, family member, etc.) provided a comprehensive multi-perspective learning experience.

13. The role-playing format significantly enhanced my doctor-patient communication and humanistic care skills.

14. This exploratory teaching method effectively addressed the monotony inherent in traditional instruction.

15. The methodology encouraged active participation and proactive exploration of practical skills.

**4. Teaching Effectiveness**

16. The training significantly improved my mastery of theoretical knowledge regarding the taught diseases.

17. My clinical operation ability and procedural skills were significantly enhanced through these drills.

18. The sessions effectively improved my ability to manage clinical problems in high-stakes emergency settings.

19. The teaching method fostered core professional qualities such as teamwork and clinical reasoning.

20. Overall, I am highly satisfied with the “Script Killing” teaching model and its impact on my professional competency.
